# Efficacy of Treatments for Anorgasmia in Premenopausal Women According to Evidence-Based Practice: A Systematic Review

**DOI:** 10.1192/j.eurpsy.2023.1346

**Published:** 2023-07-19

**Authors:** T. J. Gould, S. M. Edney

**Affiliations:** Cardiff University School of Medicine, Cardiff University, Cardiff, United Kingdom

## Abstract

**Introduction:**

This review determined the effectiveness of female anorgasmia treatments in premenopausal women using a systematic search strategy. This review considers all physiological, pharmaceutical, psychological and social treatments. Thomas and Thurston (Maturitas 2016; 87 49-60) recommend a biopsychosocial approach, where subjective distress and physical factors can coexist (Brotto *et al.* JSM 2010; 586-614). Yet, methodological issues are rife e.g., obtaining representative samples and limited assessment methods. Further, reviews are narrative with limited synthesis (Marchand SMR 2021; 9(2) 194-211). Frühauf et al. (Archives of Sexual Behavior 2013; 42(6) 915-933) completed a review, but there is no account for research published after 2007 and limited follow-up assessments.

**Objectives:**

This is the first systematic review of premenopausal anorgasmia with assessment of bias for all treatments. This review is restricted to anorgasmia to better isolate interventions and exclude comorbid conditions.

**Methods:**

10 different databases were searched (2007-2021) including studies from peer-reviewed journal articles and grey literature. Results were synthesised in forest plots according to timepoints of data, alongside different treatments to determine effect size from standardised mean differences (SMD). Outcome measures included the self-reported sexual function, sexual distress and clinician observation. The SMD was used as not all scales are consistent across studies. All results given are in line with a pre-defined analysis plan.

**Results:**

Of 1388 studies screened, 15 studies (2002-2020) were analysed: study designs were mixed with mostly self-report measures. Effective treatments included Tribulus terrestris (M=3.77, p<0.01), plasma injection (M=4.48, p<0.01), and CO2 laser therapy (M=4.06, p<0.05). For psychological studies, assessment of active sexual engagement described how subjects felt more aware of their sexuality which improved outcomes. Limitations of most studies included a very high risk of bias, notably in randomisation of subjects, allocation and outcomes. All interventions had a significant effect in independent t-tests, yet synthesis of SMDs show insignificant effect, implying data is inconclusive.

**Conclusions:**

This review aimed to systematically appraise all treatments for orgasmic satisfaction for premenopausal women. Higher levels of significance were observed for treatments across all modalities. The efficacy of natural supplements has been disputed (IsHak *et al.* JSM 2010; 7(10) 3254-3268), but this review shows promise. All psychological results provided insight into the role of the therapist-client relationship and reappraisal of traumatic sexual experience. Yet, risk of bias is likely impacted by difficulty establishing standardised scientific protocol. Considerations for future research include clear statements of randomisation and multi-faceted outcome measures.

**Disclosure of Interest:**

None Declared

